# Supplementary material for: Implementation, delivery, and utilization of iron fortified rice supplied through public distribution system across different states in India: An exploratory mixed-method study
Source: PLOS Glob Public Health. 2024 Aug 7;4(8):e0003533. doi: 10.1371/journal.pgph.0003533 (PMC11305529; doi:10.1371/journal.pgph.0003533)
Supplement: S1 Table — (DOCX) [file pgph.0003533.s001.docx]

**S1 Table**. List of pilot districts where the Centrally Sponsored Pilot Scheme on Fortification of Rice and its distribution under Public Distribution System was implemented from 2020 to 2021

| S. No. | State | District | Fortified Rice distribution Starting month |
| --- | --- | --- | --- |
| 1 | Maharashtra | Gadchiroli | February, 2020 |
| 2 | Gujarat | Narmada | February, 2020 |
| 3 | Andhra Pradesh | Vizianagaram | April, 2020 |
| 4 | Tamil Nadu | Tiruchirapally | October, 2020 |
| 5 | Chhattisgarh | Kondagaon | October, 2020 |
| 6 | Uttar Pradesh | Chandauli | January, 2021 |
| 7 | Odisha | Malkangiri | July, 2021 |
| 8 | Telangana | Jayshankar Bhupalapally | September, 2021 |
| 9 | Uttarakhand | Udam Sing Nagar | September, 2021 |
| 10 | Madhya Pradesh | Singrauli | September, 2021 |
| 11 | Jharkhand | East Singhbhum | October, 2021 |
