# Supplementary material for: Implementation, delivery, and utilization of iron fortified rice supplied through public distribution system across different states in India: An exploratory mixed-method study
Source: PLOS Glob Public Health. 2024 Aug 7;4(8):e0003533. doi: 10.1371/journal.pgph.0003533 (PMC11305529; doi:10.1371/journal.pgph.0003533)
Supplement: S1 File — (DOCX) [file pgph.0003533.s002.docx]

**In depth interview guide for District level officials**

1. Is the fortified rice made available through PDS in this district?

2. If yes, to Q1, when did the fortified rice supply through PDS start in your district?

3. Is the fortified rice through PDS is in entire district or few selected blocks/Mandals of the district?

4. Can you briefly tell us about the supply mechanism of fortified rice supplied through PDS?

*How regularly the fortified rice supply through PDS in your district?*

*Who raises demand?*

5. From where will you get the fortified rice to be supplied through PDS in your district? How frequently you will get the fortified rice?

6. How is the fortified rice supplied from district level to the fair price shops and beneficiaries?

7. How frequently do you send fortified rice from district level to peripheral Fair price shops?

8. Do you test the quality of fortified rice supplied to the district? If yes, how?

9. What is your experience in distribution of fortified rice compared to unfortified rice in the district?

*Any training provided regarding usage of fortified rice?*

*Did you receive any feedback from public?*

*Did you receive any feedback from FPS owners?*

*Is it acceptable by the beneficiaries?*

10. What do you do if fortified rice is not available in your district?

**In depth interview guide for FCI Godown Managers**

1. Is the fortified rice made available through PDS in this district?

2. If yes, to Q1, when did the fortified rice supply through PDS start in your district?

3. Is the fortified rice through PDS is in entire district or few selected blocks/Mandals of the district?

4. Does FCI godowns stock enough fortified rice to supply through PDS? Are there any issues related to the supply of Fortified rice to and fro from FCI godowns?

5. From where will you get the fortified rice to be supplied through PDS in your district? How frequently you will get the fortified rice?

6. Where do you store the fortified rice to be supplied through PDS in your district? What precautions are followed during the storage?

7. How frequently do you send fortified rice from district level to peripheral Fair price shops?

8. What do you do if fortified rice is not available in your district?

**In depth interview guide for Fair Price shop owners**

1. How many families are registered under your FPS?

2. How frequently do you distribute rice for families registered under your FPS? How many kilograms you are distributing to each person?

3. From where do you receive the rice for distribution?

4. Did you notice any change in the variety of rice that is provided to you? If so, what it is?

5. Are you getting the supply of Fortified rice (local name)?

*Is all the rice you receive is fortified or do you sometimes get other type of rice too?*

6. How do you test if the rice supplied is fortified or not? Or do you check every time the rice is supplied to you?

7. What are your experiences in distribution of fortified rice compared to unfortified rice? Or Are all the beneficiaries taking this rice or are there any issues in distribution of this rice compared to the earlier variety you used to get?

*Did you receive any positive or negative feedback?*

8. Do all the HH’s registered under your FPS take rice or is there any a reduction in the number of beneficiaries?

Any other fortified products? (Wheat flour (fortified or not fortified))

**Questionnaire for Beneficiaries**

| **S. No.** | **Household proforma** | **Code** | **Response** |
| --- | --- | --- | --- |
|  | Date of Data Collection |  |  |
|  | District Name |  |  |
|  | Block Name |  |  |
|  | Village Name |  |  |
|  | Type of Ration Card | 1. Regular PDS card  2. AAY  Others, Specify |  |
|  | Name of the Head of Household |  |  |
|  | Gender of Head of Household |  |  |
|  | Name of the respondent |  |  |
|  | Type of Family | 1. Nuclear………..01  2. Joint……………02  3. Extended Nuclear…03 |  |
|  | Total number of family members (including children) | 1. Two……….01  2. Three………..02  3. Four…………03  4. Five………….04  5. Six……………05  6. Seven………….06  7. Eight……………07  8. Nine……………08  9. Ten-----------09  10. More than ten (specify)……… |  |
|  | Source of rice for your family in the previous one year? | 1. From their farms  2. From Market  3. PDS |  |
|  | What do you receive from PDS? | 1. Rice  2. Wheat flour  3. Rice and wheat flour |  |
|  | Frequency of Rice received in last one year from PDS? | 1.Once  2. Two times  3. 3-6 times  4. Every month  5. Never |  |
|  | How do you receive the rice through PDS? | 1. By going to FPS  2. Home delivery in Vehicle |  |
|  | How did you receive the rice supplied through PDS? (Mode of packaging of Rice) | 1. In sealed pack  2. In sack brought from FPS/In your own bag  3. Other pack |  |
|  | Amount of Rice received (Kg) last month through PDS in your family? |  |  |
|  | Use of PDS Rice | 1. Consumed fully  2. Consumed partially  3. Sold  4. Did not consume at all  5. others…explain |  |
|  | Amount of PDS Rice consumed by the household members in the last month? |  |  |
|  | While washing the rice, does some rice kernels float over water? | 1. Yes  2. No |  |
|  | If Response to Q. 19 is Yes, what do you do with the floating rice kernels? | 1. Throw away  2. Use them for cooking |  |
|  | In the recent past, did you notice any change in the rice you receive? | Color 1. Yes 2. No  Texture 1. Yes 2. No  The way it cooks 1. Yes 2. No  Taste 1. Yes 2. No |  |
|  | Apart from rice, what else do you receive through PDS? |  |  |
