# Supplementary material for: Implementation, delivery, and utilization of iron fortified rice supplied through public distribution system across different states in India: An exploratory mixed-method study
Source: PLOS Glob Public Health. 2024 Aug 7;4(8):e0003533. doi: 10.1371/journal.pgph.0003533 (PMC11305529; doi:10.1371/journal.pgph.0003533)
Supplement: Attachment — Submitted filename: Response to Reviewers.docx [file pgph.0003533.s003.docx]

***PLOS Global Public Health***

**Cover letter with response to reviewer’s comments**

___________________________________________________________________________

Dear Johanna Pruller,

Thank you for kindly reviewing the manuscript titled **“Implementation, delivery, and utilization of iron fortified rice supplied through public distribution system across different states in India: An exploratory mixed-method study”** (EMID:39e43f9fdabb16d8).

We have addressed the reviewers’ comments and indicated the line and page numbers in the manuscript where the changes have been made. The changes made are also shown in the ‘track change’ version.

This manuscript/material has not and will not be offered elsewhere for possible publication, as long as it is under consideration in PLOS Global Public Health.

Thanking you,

Samarasimha Reddy

Dr Samarasimha Reddy N

Scientist ‘E’ (Medical),

Clinical Epidemiology Division,

ICMR-National Institute of Nutrition,

Hyderabad – 500007, India

E-mail: [drsamarareddy@gmail.com](mailto:drsamarareddy@gmail.com)

_____________________________________________________________________________

| **Sl. No.** | **Reviewer’s/editorial comments** | **Correction/revision done in Track Changes Mode): Line/Table number as per the Track Changes file** | **Page/s** |
| --- | --- | --- | --- |
| **Journal Requirements:** | | | |
| 1. | Please review your reference list to ensure that it is complete and correct. If you have cited papers that have been retracted, please include the rationale for doing so in the manuscript text, or remove these references and replace them with relevant current references. Any changes to the reference list should be mentioned in the rebuttal letter that accompanies your revised manuscript. If you need to cite a retracted article, indicate the article’s retracted status in the References list and also include a citation and full reference for the retraction notice. | We have reviewed the reference list and confirm that it is complete and correct. We have added references 12-16, 20, 32, and 33 and highlighted in yellow in the text as well as the reference list.  Line Numbers: 74, 90, 536 | 3, 4, 24 |
| 2. | Please amend your detailed Financial Disclosure statement. This is published with the article. It must therefore be completed in full sentences and contain the exact wording you wish to be published.  a. State the initials, alongside each funding source, of each author to receive each grant.  b. State what role the funders took in the study. If the funders had no role in your study, please state: “The funders had no role in study design, data collection and analysis, decision to publish, or preparation of the manuscript.”  c. If any authors received a salary from any of your funders, please state which authors and which funders.  If you did not receive any funding for this study, please simply state: “The authors received no specific funding for this work. | Revised as suggested (lines: 653) | 28 |
| 3. | Please send a completed 'Competing Interests' statement, including any COIs declared by your co-authors. If you have no competing interests to declare, please state "The authors have declared that no competing interests exist". Otherwise please declare all competing interests beginning with the statement "I have read the journal's policy and the authors of this manuscript have the following competing interests:" | We have incorporated this in the lines 639-641 | 28 |
| 4. | Please provide separate figure files in .tif or .eps format only and remove any figures embedded in your manuscript file. Please also ensure all files are under our size limit of 10MB.  For more information about figure files please see our guidelines:  https://journals.plos.org/globalpublichealth/s/figures  <https://journals.plos.org/globalpublichealth/s/figures#loc-file-requirement> | Figures are provided as per the prescribed guidelines. |  |
| 5. | In the online submission form, you indicated that "The data is available on request to corresponding author". All PLOS journals now require all data underlying the findings described in their manuscript to be freely available to other researchers, either 1. In a public repository, 2. Within the manuscript itself, or 3. Uploaded as supplementary information.  This policy applies to all data except where public deposition would breach compliance with the protocol approved by your research ethics board. If your data cannot be made publicly available for ethical or legal reasons (e.g., public availability would compromise patient privacy), please explain your reasons by return email and your exemption request will be escalated to the editor for approval. Your exemption request will be handled independently and will not hold up the peer review process, but will need to be resolved should your manuscript be accepted for publication. One of the Editorial team will then be in touch if there are any issues. | The data collected for the study include sensitive information about participants (district supply officers, fair price shop dealers and beneficiaries of public distribution system) and their opinion towards the government’s policies and programs and various welfare schemes/services. Despite deidentification of the data, markers can be picked up from the transcripts through which the identity of the participants may be compromised. Therefore, sharing of data publicly will not be appropriate, as this information will put the participants at risk of being identified. Additionally, the institutional ethics committee of the Indian Council of Medical Research – National institute of Nutrition, Hyderabad, India doesn’t allow publicly sharing of data for publication purpose. However, there is a provision for the bonafide researchers to access the de-identified data after seeking prior approvals from the Principal Investigators (Dr. Samarasimha Reddy N; Email: [drsamarareddy@gmail.com](mailto:drsamarareddy@gmail.com) / Dr. Hemant Mahajan; Email: [Hemant.mahajan.84@gmail.com](mailto:Hemant.mahajan.84@gmail.com) ) and the Member secretary of the ethics committee Indian Institute of Public health, Hyderabad, India (Dr. Uday Kumar, Email ID: putchaaudaykumar@yahoo.com). |  |
| 6. | We have noticed that you have uploaded Supporting Information files, but you have not included a list of legends. Please add a full list of legends for your Supporting Information files after the references list. | Revised as suggested in lines 762-765 | 31 |
| 7. | Figures 1 & 2: Please confirm (a) that you are the photographer; or (b) provide written permission from the photographer to publish the photo(s) under our CC-BY 4.0 license. | We confirm that the photographs are taken by the authors of the manuscript who provide their permission to publish the photos. |  |
| **Reviewer #1** | | | |
| 1. | The strategy evaluated is undoubtedly important. However, it has been published that social group is important for the prevalence of anemia in women [Sharif, N., Das, B., & Alam, A. (2023). Prevalence of anemia among reproductive women in different social group in India: Cross-sectional study using nationally representative data. PloS one, 18(2), e0281015. ], undernutrition is related to anemia in children [Stiller, C. K., Golembiewski, S. K. E., Golembiewski, M., Mondal, S., Biesalski, H. K., & Scherbaum, V. (2020). Prevalence of Undernutrition and Anemia among Santal Adivasi Children, Birbhum District, West Bengal, India. International journal of environmental research and public health, 17(1), 342. https://doi.org/10.3390/ijerph17010342], and anemia is disproportionately concentrated in low socioeconomic groups in low-income and middle-income countries [Balarajan, Y., Ramakrishnan, U., Ozaltin, E., Shankar, A. H., & Subramanian, S. V. (2011). Anaemia in low-income and middle-income countries. Lancet (London, England), 378(9809), 2123–2135. https://doi.org/10.1016/S0140-6736(10)62304-5]. Maybe it is not polite to remember the reality: poverty leads to anemia. Iron deficiency anemia in India is an old problem [Anand, T., Rahi, M., Sharma, P., & Ingle, G. K. (2014). Issues in prevention of iron deficiency anemia in India. Nutrition (Burbank, Los Angeles County, Calif.), 30(7-8), 764–770. https://doi.org/10.1016/j.nut.2013.11.022]. Onyeneho considers that "Combining nutritional supplementation and food-fortification programmes with reduction in maternal anemia and family poverty may yield optimal improvement of childhood anemia in India." [Onyeneho, N. G., Ozumba, B. C., & Subramanian, S. V. (2019). Determinants of Childhood Anemia in India. Scientific reports, 9(1), 16540. https://doi.org/10.1038/s41598-019-52793-3]. Authors could explain a little bit more why they did not profounder regarding poverty. Poverty is related with low education, undernutrition, an some other problems previous to develop anemia. It is a strength the study was conducted in six districts of different states, but it would be better some kind of stratification of population. It is known that one of the weaknesses of qualitative studies is the response bias to interview questionnaires, so authors could use population hemoglobin registries to correlate with potential benefits of the utilization of iron fortified rice supplied by government. | Thank you very much for the valuable and extensive comment. We have referred all the citations provided in the comment to improve our background. We agree that poverty is very important in terms of anemia. However, the impact of poverty on anemia is beyond the scope of our study which included only the implementation, delivery, and utilization of iron fortified rice supplied through public distribution system as a formative study. Based on the findings of the formative research, we have begun an impact evaluation study “Impact Evaluation of the Iron Fortified Rice Supplied through public distribution system and other welfare schemes in reducing the Burden of Iron deficiency Anemia in India” that would shed light on the effect of fortified rice as well as poverty and wealth on anemia. Also, the Targeted Public Distribution System (TPDS) supply commodities to the economically weaker sections of the population. |  |
| **Reviewer #2** | | | |
| 1. | This paper reports on interviews and surveys from a range of stakeholders in India’s public distribution of fortified rice. The program is ambitious and the stakeholders seem largely positive about its success. This seems like valuable information to get into the published literature, and I offer some suggestions for how the authors might make minor revisions to make their work more accessible to a wider range of readers.  Line 74: Folic acid --- also very valuable, especially for prevention neural tube defects, and perhaps best delivered through fortified foods. Worth emphasizing more. | Revised as suggested. This has been incorporated in the lines ___ which reads as below:  Fortification of rice with folic acid, in addition to preventing megaloblastic anemia, could have the additional benefit for protecting against the development of neural tube defects by delivering the vitamin to women before and during pregnancy [12–16]. |  |
| 2. | Line 90: did the Cochrane review report a confidence interval for the mean difference of 109? If so, you should include it, too. | No. The Absolute reduction of 109 per 1000 was calculated by subtracting 279 per 1000 (risk of anemia with fortified rice) from 388 per 1000 (risk of anemia with unfortified rice). |  |
| 3. | Line 125: you could explain here what exactly a “fair price shop” is, for readers who are not familiar (you provide a bit more detail on Line 341-342, but I would be interested to know even more, and sooner) | Revised as suggested. They are incorporated in the lines: 71-74 in the revised manuscript which reads as below:  Public Distribution system is a supplemental food security system for providing foodgrains at affordable prices in India. They provide rice, wheat, sugar, and kerosene. In some states, other items such as pulses, iodized salt, and edible oil are also provided. These commodities are provided through the Fair Price Shops (FPS), which are also known as Ration shops [20]. | 3 |
| 4. | Line 129: conducting all of these interviews in five days seems impossible! Are you sure the May 1 to 5, 2023 time period is correct? | Yes, the time period given is correct. Six teams consisting of public health/medical professionals and a biomarker expert were formed. Each team visited a state mentioned in the study simultaneously. The teams took five days to conduct the interviews from May 1-5, 2023. We made sure that each team comprised of members with expertise in qualitative methodologies. All the interviewers before conducting the interviews have gone through standardized training. |  |
| 5. | Line 142 / Fig 1: I found this figure distracting. Perhaps a table would be a more effective way to share this information. | Revised as suggested (Table 1; lines 152-156) | 7 |
| 6. | Line 143-147: consider specifying here which were qualitative interviews and which were quantitative. If I understand correctly, the 181 beneficiaries provided quantitative responses to a structured interview while all the rest were unstructured interviews with qualitative analyses. | Revised as suggested in the lines 149-151 which reads as below:  The beneficiaries provided quantitative responses to the structured interview questionnaire, while in-depth interviews were conducted with the other stakeholders. | 7 |
| 7. | Line 150-157: I would be interested in a summary table and/or some examples of these constructs. | Thank you very much for suggesting this. We would like to present these constructs as it is and the examples are mentioned as verbatims in the results section. |  |
| 8. | Line 171-172: I was surprised that you needed to take special care to avoid interviewing the same respondents for the qual and quant parts. Did I misunderstand something? | The beneficiaries provided quantitative responses to the structured interview questionnaire, while in-depth interviews were conducted with the other stakeholders. To prevent bias, we did not include DSO, FPS Dealer, or Warehouse supervisors in the category of beneficiaries. |  |
| 9. | Line 182-183: I suggest you include your questionnaires as supplementary material. | Questionnaire has been incorporated as Supplementary File 2. |  |
| 10. | Line 185: here is says you had 36 unstructured interviews, but in Figure 1 you say 52. I think these should be the same number. | We have conducted 52 unstructured and 181 structured interviews. We have revised the number and incorporated in line 194 | 9 |
| 11. | Figure 2: I don’t know if this figure add much, I found it distracting. | We have discussed internally with co-investigators and we strongly feel that we should keep the figure as it was directly provided by one of the stakeholders to inform local system for distribution of fortified rice through fair price shops. To avoid the confusion, we have given footnotes for the figure to fully explain rice acquisition and fortified rice distribution. |  |
| 12. | Line 256: the IEC activities seem important! Do you have more detail from your qual analysis that you can include about them? | Yes, we have incorporated them in the lines 266-269 which reads as below:  The IEC activities included posters and banners in FPS shops, demonstrations with live experiments to remove the myth of “plastic rice” by burning the rice, interactive talks carried out by experts (for example, experts from Food Research Laboratory and Nutrition International in Gujarat). | 11 |
| 13. | Line 441: I would like more information about your approach to direct observation. What was the protocol? How many subjects were observed? How did you have time to do this in only five days of data collection? | We have observed rice cooking practices in one house per state. So, we observed total six houses for rice cooking across six states. We mainly looked for rice washing practices and FRK floating before cooking as well as water decanting practices. We also observed changes in color or texture of the cooked rice. Six teams consisting of public health/medical professionals and a biomarker expert were formed. Each team visited a state mentioned in the study simultaneously. |  |
| 14. | Line 469: Table 1 shows a high level of “clustering” by location. I would be interested to know more about the reasons behind this, and it could be a more useful way to organize the information you currently have presented in Table 1. | There could be multiple reasons behind this such as state level differences such as differences in the supplier of FRk, common cooking practices in a locality, information, education, and communication activities, cultural influences, and interviewer bias. |  |
| 15. | Line 493: what is the scale of acceptance rating? Is 6.9 high acceptance? | The scale of acceptance rating is based on Lawless and Heymann hedonic scale (https://link.springer.com/book/10.1007/978-1-4419-6488-5). A rating of 6.9-7.4 comes under the category of “Like Moderately”. In sensory evaluation studies, a score of 7 or higher is generally considered indicative of highly acceptable sensory quality (https://www.sciencedirect.com/science/article/abs/pii/B9780123741240000089) |  |
| 16. | Line 500: it seems like your work has identified at least three important threats to success in fortification: (1) rejecting the product (2) picking out the FRK (3) decanting the liquid. You should include a table or figure to emphasize these. Are there other important threats that you discovered? | Revised as suggested in Table 3; lines 525-526 | 23, 24 |
| 17. | Line 519: I am not familiar with the term of art “micronized FPP”. Consider including a reference for other readers who are unfamiliar and want to learn more. | Revised as suggested. We have added two references:  1. Fidler MC, Walczyk T, Davidsson L, Zeder C, Sakaguchi N, Juneja LR, et al. A micronised, dispersible ferric pyrophosphate with high relative bioavailability in man. Br J Nutr. 2004;91: 107–112. doi:10.1079/BJN20041018  2. Radhika MS, Nair KM, Kumar RH, Rao MV, Ravinder P, Reddy CG, et al. Micronized ferric pyrophosphate supplied through extruded rice kernels improves body iron stores in children: a double-blind, randomized, placebo-controlled midday meal feeding trial in Indian schoolchildren. Am J Clin Nutr. 2011;94: 1202–1210. doi:10.3945/ajcn.110.007179  These are incorporated in the lines: 535-536 | 24 |
|  |  |  |  |
